# Bridging the Perception–Performance Gap in Scientific Literacy: Evidence from Junior High School Students’ Self-Reports and Objective Assessment

**DOI:** 10.12688/f1000research.189469.1

**Published:** 2026-09-11

**Authors:** Megawati Megawati, Muhiddin Palennari, Khaeruddin Khaeruddin, Halimah Husain, Ahmad Swandi

**Affiliations:** 1 Doctoral Program in Educational Sciences, Postgraduate Program, State University of Makassar, Makassar, South Sulawesi, Indonesia; 2 Biology Education Study Program, State University of Makassar, Makassar, South Sulawesi, Indonesia; 3 Physics Education Study Program, State University of Makassar, Makassar, South Sulawesi, Indonesia; 4 Chemistry Education Study Program, State University of Makassar, Makassar, South Sulawesi, Indonesia; 5 Science Education Study Program, Bosowa University, Makassar, South Sulawesi, Indonesia

**Keywords:** scientific literacy; perceived competence; demonstrated performance; self-assessment; junior high school; science education; perception–performance gap

## Abstract

**Background:**

Scientific literacy involves not only students’ knowledge of scientific concepts but also their ability to explain phenomena, interpret scientific information, draw conclusions, and design investigations. However, students’ perceptions of their own scientific competence may not always correspond to what they can demonstrate through objective assessment. Examining both perspectives can provide a more comprehensive understanding of students’ scientific literacy and learning needs.

**Purpose:**

This study aimed to examine junior high school students’ perceived and demonstrated scientific literacy and to identify discrepancies between the two across directly comparable domains.

**Methods:**

A descriptive comparative design was employed with 30 junior high school students from two schools in Luwu Regency, South Sulawesi, Indonesia, selected through purposive sampling. Data were collected using the Junior High School Science Learning Needs and Initial Profile Questionnaire, which examined students’ science learning interest, project-based learning experience, and perceived scientific literacy. Students also completed a 15-item objective scientific literacy assessment scored dichotomously. Descriptive analyses were conducted, followed by domain-level comparisons across scientific explanation, data/graph/table interpretation, conclusion drawing, and experimental/investigation design.

**Results:**

Students demonstrated generally positive engagement with science learning and substantial experience with project-based learning. Their overall perceived scientific literacy was 68.50%, whereas demonstrated scientific literacy was 39.80%, indicating a 28.70-percentage-point discrepancy. Across the four comparable domains, perceived competence consistently exceeded demonstrated performance. The largest discrepancy occurred in conclusion drawing, followed by experimental/investigation design and scientific explanation, while data/graph/table interpretation showed the smallest discrepancy.

**Conclusion:**

The findings indicate that students’ perceived scientific literacy provides a more favorable picture than their demonstrated performance, particularly for higher-order scientific reasoning competencies. Combining self-report and objective assessment can therefore provide a more informative basis for identifying students’ scientific literacy needs. Science instruction should place greater emphasis on evidence-based conclusion drawing, experimental reasoning, scientific explanation, and students’ ability to reflect on their actual performance.

## Introduction

Scientific literacy has become an important goal of science education because students are increasingly expected not only to understand scientific concepts but also to use scientific knowledge when interpreting phenomena, evaluating information, and making reasoned decisions ( Gormally et al., 2012; Roy et al., 2025). In this perspective, learning science involves more than the acquisition of factual knowledge. It requires students to mobilize scientific knowledge in situations that may be unfamiliar, interpret evidence, evaluate explanations, and use scientific reasoning to address problems encountered in everyday life ( Evains et al., 2024; Laugksch, 2000; Valladares, 2021).

The OECD framework has consistently positioned scientific literacy as the capacity to engage with science-related issues and ideas through reasoned scientific discourse ( Harlen, 2001). Within the PISA framework, this competence encompasses explaining phenomena scientifically, evaluating and designing scientific enquiry, and interpreting data and evidence scientifically. The more recent PISA 2025 framework further emphasizes the importance of constructing and evaluating scientific enquiry designs, critically interpreting scientific data and evidence, and evaluating and using scientific information for decision making and action ( OECD, 2022; Tytler et al., 2025).

This conception has important implications for how students’ scientific literacy should be understood and assessed. A student may know relevant scientific concepts yet still experience difficulty when required to interpret a graph, evaluate the adequacy of an investigation, distinguish evidence from unsupported claims, or draw a conclusion that is consistent with available data ( Bhaw et al., 2023; Woolley et al., 2018). Scientific literacy therefore represents an integrated competence involving conceptual, procedural, and epistemic dimensions of scientific knowledge rather than a single measure of content mastery ( Osborne & Allchin, 2025).

One challenge in science education concerns how students’ perceptions of their own competence correspond to what they can actually demonstrate. Students’ judgments about their learning are valuable because they provide information about how learners perceive their strengths, weaknesses, and progress ( Carpenter et al., 2020). Self-assessment is also closely associated with reflection and self-regulated learning, making students’ perceptions potentially useful for understanding learning processes ( Arvatz et al., 2025; Yan, 2020). However, self-reported competence should not automatically be treated as equivalent to demonstrated competence. Research on student self-assessment has repeatedly shown that the correspondence between students’ judgments and external performance criteria can be variable. Andrade (2019) critical review, for example, notes that research on self-assessment has examined its relationship with achievement and the consistency between students’ judgments and other forms of assessment, while emphasizing that self-assessment should be interpreted carefully rather than assumed to provide a direct measure of actual performance.

The issue becomes particularly important when the competence being assessed involves complex reasoning. Students may recognize that they understand a scientific topic or believe that they can perform a particular scientific task without necessarily being able to apply that understanding successfully in a new situation. Studies of calibration have shown that learners can differ substantially in the accuracy of their judgments about their own performance, with discrepancies particularly evident among lower-performing learners ( Nepal & Kafle, 2024). A large meta-analytic review likewise indicates that self-assessment accuracy is an important but imperfect aspect of student learning and should be examined in relation to an appropriate performance criterion.

This distinction suggests that examining students’ perceived scientific literacy and their demonstrated scientific literacy separately may provide only a partial picture of their learning. Self-reports can reveal how students understand their own capabilities, whereas objective assessments provide evidence of what they can demonstrate under specified task conditions ( Gabbard & Romanelli, 2021). Considering both forms of evidence may therefore provide a more informative diagnosis of students’ scientific literacy than relying on either source alone.

The distinction between perceived and demonstrated competence is also relevant to contemporary approaches to science learning that emphasize active participation. Project-based learning, for example, has received considerable attention in science education because it provides opportunities for students to investigate problems ( Pozuelo-Muñoz et al., 2023), collaborate (Mitha Dwi Anggriani et al., 2024), apply concepts ( Swandi et al., 2022), produce products ( Martawijaya et al., 2023), and connect learning with authentic situations ( Burhan et al., 2026). Reviews of the literature have reported promising relationships between project-based learning and the development of scientific literacy.

Nevertheless, participation in project activities should not automatically be interpreted as evidence that all dimensions of scientific literacy have been developed. Scientific literacy requires students to engage with evidence and reasoning throughout the learning process. A project may involve collaboration and production of a tangible outcome while providing limited opportunities for students to interpret data critically, evaluate the quality of evidence, justify claims, or reflect on the validity of conclusions. From this perspective, the educational value of project-based learning depends not only on whether students participate in projects but also on the kinds of cognitive and epistemic processes embedded within those projects.

This issue is particularly relevant to the assessment of scientific literacy. The competencies emphasized in contemporary science frameworks require students to move between scientific representations, interpret evidence, evaluate investigation designs, and construct conclusions that are warranted by available information. Consequently, students’ experiences in active or project-based learning should be considered alongside direct evidence of their ability to perform these scientific reasoning processes.

Although self-report measures and objective assessments serve different purposes, examining them together offers an opportunity to identify areas in which students’ perceived competence and demonstrated performance may not align. Such discrepancies are educationally meaningful because they can reveal dimensions of scientific literacy that students may not yet recognize as areas requiring further development. Conversely, alignment between perceived and demonstrated competence may indicate that students have a relatively well-calibrated understanding of their own abilities ( Gabbard & Romanelli, 2021).

The concept of calibration is particularly useful in this regard. Calibration refers to the correspondence between learners’ perceptions of their performance and their actual performance on a task. Research in science education has demonstrated that students’ self-evaluations are not always closely aligned with their demonstrated performance, and that the quality of this alignment can vary according to students’ level of achievement and learning experience ( Batool & Bagum, 2025; Dorfman & Fortus, 2019). Thus, examining the two sources of evidence together is not simply a matter of comparing two scores; it provides insight into how students understand their own scientific capabilities in relation to what they can actually demonstrate.

For science educators, this distinction has practical significance. If students’ perceived competence is higher than their demonstrated ability in particular aspects of scientific reasoning, instruction may need to provide more explicit opportunities for students to examine evidence, justify conclusions, interpret representations, and reflect on the quality of their own reasoning. Conversely, if students underestimate abilities that they can demonstrate successfully, instructional responses may need to address confidence and metacognitive awareness. In both cases, the comparison between perception and performance can contribute to a more precise diagnosis of learning needs.

Despite the growing literature on scientific literacy assessment and student self-assessment, these two perspectives are often examined independently. Scientific literacy studies commonly emphasize performance on structured assessment tasks, whereas research on self-assessment tends to focus on students’ perceptions, metacognitive processes, or the effects of self-assessment on learning. The two perspectives are therefore not always integrated to examine whether students’ perceptions of their scientific capabilities correspond with their demonstrated performance in the same learning context.

This gap is particularly important at the junior high school level, where students are developing more sophisticated forms of scientific reasoning and are increasingly expected to move beyond recalling concepts toward interpreting evidence, evaluating explanations, and designing or evaluating investigations. A closer examination of perceived and demonstrated scientific literacy can therefore provide a more nuanced understanding of students’ learning needs and identify specific competencies that may require greater instructional attention.

Against this background, the present study examines students’ perceived and demonstrated scientific literacy using two complementary sources of evidence: a self-report questionnaire and an objective scientific literacy assessment. The study specifically seeks to identify the extent to which students’ perceptions of their scientific literacy correspond with their demonstrated performance and to determine which aspects of scientific literacy warrant particular instructional attention.

The study addresses the following research questions:

1. What is the level of junior high school students’ perceived scientific literacy based on self-reported indicators?
2. What is the level and distribution of students’ demonstrated scientific literacy based on an objective assessment?
3. What similarities and discrepancies emerge between students’ perceived and demonstrated scientific literacy?

By addressing these questions simultaneously, the study aims to contribute to a more comprehensive diagnosis of scientific literacy in junior high school science education. Rather than treating students’ self-reports and test performance as interchangeable measures, the study considers them as complementary forms of evidence that can illuminate different dimensions of students’ scientific learning.

## Methods

### Research design

This study employed a descriptive comparative quantitative design to examine junior high school students’ scientific literacy from two complementary perspectives: their perceived competence and their demonstrated performance. The design was selected because the study did not involve an instructional intervention or an experimental comparison, but rather sought to describe students’ existing scientific literacy and examine the extent to which self-reported perceptions corresponded with performance on an objective assessment.

Two sources of quantitative evidence were used. The first was a self-report questionnaire designed to capture students’ perceptions of their perceived scientific literacy, together with their interest in science learning and experience with project-based learning. The second was an objective scientific literacy test consisting of contextual multiple-choice questions designed to assess students’ ability to apply scientific knowledge and reasoning. The two sources were analyzed separately and subsequently considered together to identify patterns of convergence and discrepancy between perceived and demonstrated scientific literacy.

This design allowed the study to distinguish between what students perceived they were able to do and what they were able to demonstrate when responding to scientific problems. Such a distinction was central to the purpose of the study and provided the basis for examining the perception–performance relationship without assuming that the two measures represented identical constructs or were directly interchangeable.

### Participants

This research was conducted from September 2025 to February 2026. The participants were 30 junior high school students from two junior high schools in Luwu Regency, South Sulawesi, Indonesia, selected using purposive sampling based on their relevance to the research context and the objectives of the study. The students were approximately 13–14 years old, comprising 15 male and 15 female students. All selected students completed both the scientific literacy self-report questionnaire and the objective scientific literacy assessment.

The questionnaire collected basic participant information, including initials, school, grade level, and gender. Before completing the questionnaire, students were informed that their responses would be treated confidentially and used solely for research purposes. The same participants subsequently completed the objective scientific literacy assessment. Because the study focused on examining the correspondence between students’ perceived scientific literacy and their demonstrated performance, the analysis included only participants who provided complete data for both instruments. The final dataset therefore comprised 30 student responses for the self-report questionnaire and 30 corresponding responses for the objective scientific literacy assessment.

### Ethical considerations and informed consent

The study was approved by the Institutional Review Board at Universitas Bosowa on 4 July 2025 (Reference No. 174b/1.02/DRIPM-UNIBOS/VII/2025). The student participants were minors aged approximately 13–14 years. In accordance with the ethical procedure reviewed and approved by the Institutional Review Board, verbal informed consent was obtained from the parents or legal guardians of the participating students. Parents or legal guardians were informed verbally about the study, including its purpose, procedures, and their children’s participation. Verbal consent was used because many parents or legal guardians lived at considerable distances from the participating schools and had limited access to written or electronic communication, making the collection of physical or electronic consent forms impractical. The Institutional Review Board reviewed the study protocol and considered the consent procedure appropriate for the study context. Permission to conduct the study was obtained from the participating schools and school principals, and the students voluntarily agreed to participate. Participation was voluntary, and students were informed that their responses would be treated confidentially and used solely for research purposes.

### Instruments

Data were collected using the Junior High School Science Learning Needs and Initial Profile Questionnaire and an objective scientific literacy assessment. The questionnaire consisted of three sections addressing students’ science learning interest, project-based learning experience, and perceived scientific literacy. The perceived scientific literacy section covered seven indicators: explaining scientific phenomena, connecting science concepts with everyday life, interpreting graphs or tables, drawing conclusions from experiments, designing simple experiments, distinguishing scientific from non-scientific information, and using evidence to support opinions. The objective assessment consisted of 15 multiple-choice items designed to assess students’ ability to apply scientific knowledge and reasoning in contextualized situations. The items covered scientific explanation, data or graph interpretation, conclusion drawing, and experimental or investigation design.

Both instruments underwent expert validation involving three experts in science education and/or science assessment. Each item was evaluated for its suitability and relevance to the intended construct. An item was considered valid and suitable for use when it achieved a minimum percentage score of 75% from the expert assessment. Items meeting or exceeding this criterion were retained for data collection. This validation procedure was conducted to ensure that the questionnaire items and objective assessment items were appropriate for measuring the intended aspects of students’ science learning profile and scientific literacy. Following expert validation, the finalized instruments were administered to the selected participants. The questionnaire was used to obtain students’ self-reported perceptions and learning experiences, whereas the objective assessment was used to obtain evidence of their demonstrated scientific literacy.

### Data analysis

The data analysis consisted of two stages. First, the questionnaire and objective assessment were analyzed independently to establish separate profiles of perceived and demonstrated scientific literacy. Second, the two profiles were examined together to identify areas of convergence and discrepancy.

Responses to the seven scientific literacy self-report items were summarized using mean scores and percentages. Because the questionnaire used a four-point Likert scale, the mean score for each indicator was calculated and converted into a percentage of the maximum possible score. The overall perceived scientific literacy score was calculated in the same manner.

The interpretation of the questionnaire followed the percentage categorization used in the source instrument, in which the overall perceived scientific literacy was classified according to the resulting percentage. This procedure enabled the seven indicators to be compared on a common scale and allowed the study to identify areas in which students perceived themselves as relatively stronger or weaker.

The analysis focused particularly on seven indicators of perceived scientific literacy: explaining scientific phenomena; connecting science concepts with everyday life; reading and understanding graphs or tables; drawing conclusions from experiments; designing simple experiments; distinguishing scientific and non-scientific information; and using evidence to support an opinion. For the objective test, each correct response received a score of one, while each incorrect response received a score of zero, and individual student scores were converted into percentages based on the 15 items.

Descriptive statistics were then calculated, including mean, median, minimum score, maximum score, frequency, and percentage of students within each performance category. The objective assessment classified students into four categories: Low (<40%), Moderate (40–59%), High (60–79%), and Very high (80–100%). These categories were used to describe the distribution of demonstrated scientific literacy across the participating students. The source assessment reports the resulting distribution as 12 students in the low category, 12 in the moderate category, six in the high category, and none in the very-high category.

The final stage of analysis examined the two forms of evidence comparatively. The overall percentage obtained from the self-report questionnaire was considered as an indicator of perceived scientific literacy, whereas the percentage obtained from the objective test represented demonstrated scientific literacy.

The comparison was not treated as a direct statistical comparison of two equivalent scores because the instruments differed in response format, scoring procedure, and measurement approach. Instead, the analysis focused on identifying patterns of convergence and discrepancy between students’ perceptions and their demonstrated performance.

At the indicator level, particular attention was given to competencies that appeared in both the self-report framework and the objective assessment, including scientific explanation, interpretation of data or representations, drawing evidence-based conclusions, and designing or evaluating scientific investigations. The test blueprint was used to identify the scientific reasoning demands represented by the 15 objective items.

This comparison provided the basis for identifying whether students’ perceptions were broadly consistent with their demonstrated abilities or whether particular dimensions of scientific literacy showed a notable divergence between perception and performance.

### Analytical framework

The analytical framework of the study was organized around a distinction between perceived competence and demonstrated competence ( Figure 1). Perceived competence was derived from students’ responses to the seven scientific literacy statements in the questionnaire, while demonstrated competence was derived from performance on the 15-item objective assessment. The two datasets were first examined independently and then brought together at the interpretive stage. This structure enabled the analysis to move from describing students’ self-perceptions, to establishing their demonstrated level of scientific literacy, and finally to identifying areas where the two forms of evidence converged or diverged.

**Figure 1.**
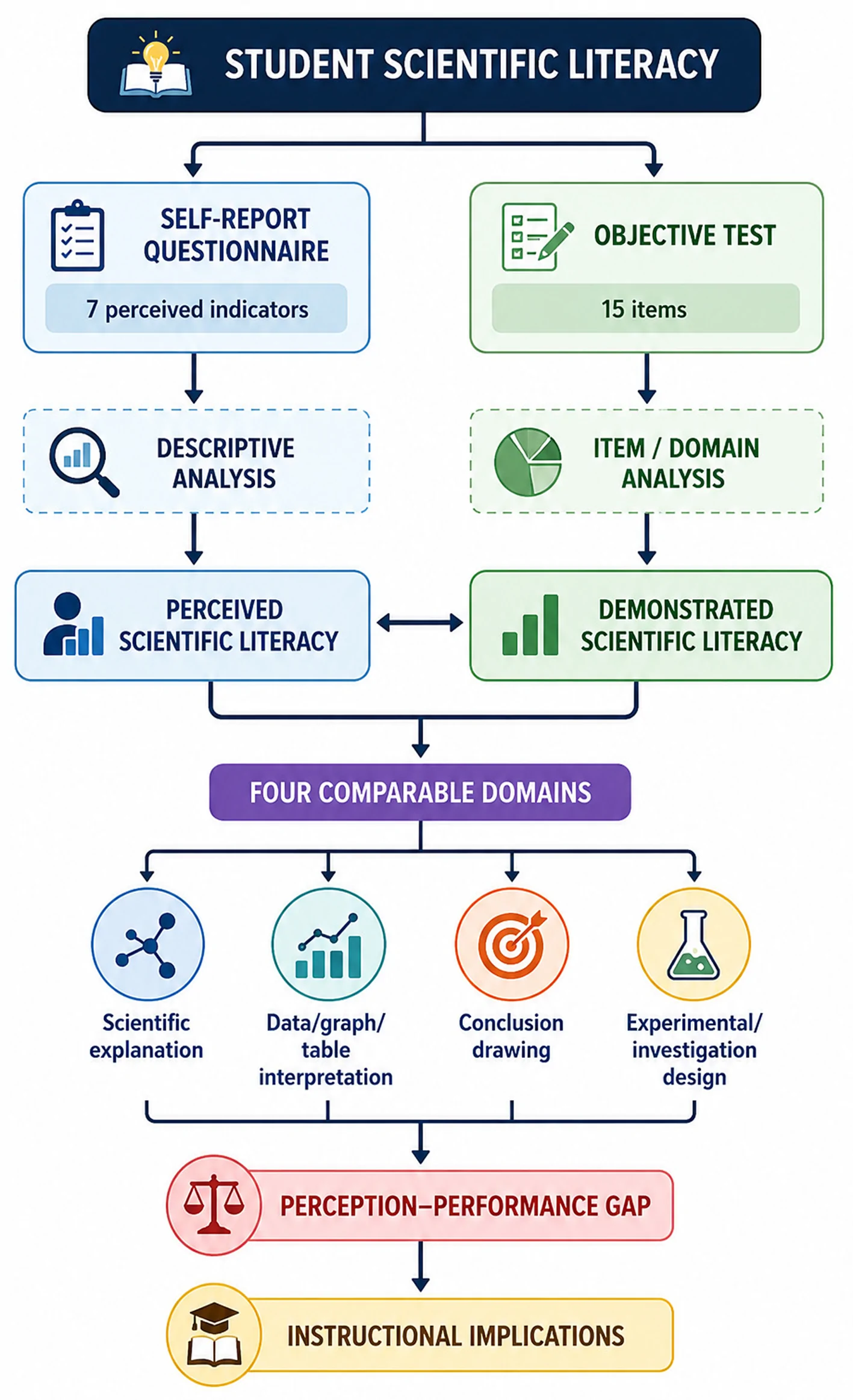
Analytical framework.

## Results

### Students’ interest in science learning

The first section of the student questionnaire examined students’ interest in science learning through six indicators: interest in learning science, enjoyment of science lessons, curiosity about natural phenomena, active participation in asking questions, efforts to seek additional information, and perceptions of the importance of science in everyday life. The questionnaire used a four-point Likert scale, with higher scores indicating stronger agreement with each statement.

Based on Table 1, the results indicate that students generally reported positive attitudes toward science learning. The highest percentage was observed for students’ enjoyment of science lessons, with a mean score of 3.80 out of 4.00 (95.00%). This was followed by the perception that science is important in everyday life, with a mean of 3.53 (88.30%), and students’ interest in learning science, with a mean of 3.40 (85.00%).

**Table 1.** Students’ interest in science learning.

No.	Indicator	Mean	Percentage
P1	Interested in learning science	3.40	85.00%
P2	Enjoy science lessons	3.80	95.00%
P3	Want to know more about natural phenomena	2.77	69.20%
P4	Actively ask questions during science lessons	2.60	65.00%
P5	Seek additional information about science	2.90	72.50%
P6	Consider science important in everyday life	3.53	88.30%

Students’ curiosity about natural phenomena reached a mean score of 2.77 (69.20%), while their efforts to seek additional information about science outside the classroom reached 2.90 (72.50%). The lowest score was found for active participation in asking questions during science lessons, with a mean of 2.60 (65.00%).

The questionnaire document summarizes these findings as generally high interest in science learning, while also identifying a difference between students’ enjoyment of science and their active participation during lessons.

### Students’ experience with project-based learning

The second section examined students’ experiences with project-based learning (PjBL) through seven indicators. These included prior experience with project-based learning, frequency of project assignments, collaboration, active involvement in projects, perceived contribution of projects to understanding science, experience presenting project results, and difficulties encountered in completing projects.

Based on Table 2, the results show that students were generally familiar with project-based learning. The proportion reporting that they had previously participated in project-based learning was 88.30%, with a mean score of 3.53. Similarly, 80.80% reported that teachers frequently assigned projects in science lessons. Students also reported relatively high levels of collaboration (83.30%) and active involvement in designing and completing projects (82.50%).

**Table 2.** Students’ experience with project-based learning.

No.	Indicator	Mean	Percentage
P1	Previously participated in project-based learning	3.53	88.30%
P2	Teachers frequently provide science projects	3.23	80.80%
P3	Collaborate with peers in completing projects	3.33	83.30%
P4	Actively design and complete projects	3.30	82.50%
P5	Projects help understand science content	3.53	88.30%
P6	Have presented project results	2.70	67.50%
P7	Experience difficulties completing projects	3.53	88.30%

Students also perceived project-based learning as useful for understanding science content. The corresponding mean score was 3.53, equivalent to 88.30%. However, the percentage of students reporting experience in presenting project results was lower, at 67.50%, with a mean score of 2.70.

The seventh item addressed difficulties in completing projects. The mean score was 3.53 (88.30%), indicating that a large proportion of students reported experiencing difficulties when completing project activities. Because this item was negatively worded in the questionnaire, it is reported here as an indicator of project-related difficulty, rather than as a positive project-learning outcome.

Overall, the questionnaire data show that students had relatively substantial exposure to project-based learning, while presentation experience and reported difficulties in completing projects represented notable features of their project-learning experience.

### Students’ perceived scientific literacy

The third section of the questionnaire assessed students’ perceived scientific literacy through seven indicators. These indicators represented students’ perceptions of their ability to explain natural phenomena scientifically, connect science concepts with everyday life, read and understand graphs or tables, draw conclusions from experiments, design simple experiments, distinguish scientific from non-scientific information, and use evidence to support their opinions.

Based on Table 3, the results show variation across the seven indicators. The highest perceived competence was found for designing simple experiments, which obtained a mean score of 3.10 out of 4.00 (77.50%). This was followed by the ability to connect science concepts with everyday life, with a mean of 3.07 (76.70%), and the ability to explain natural phenomena scientifically, with a mean of 3.03 (75.80%).

**Table 3.** Students’ perceived scientific literacy by indicator.

No.	Scientific literacy indicator	Mean	Percentage
P1	Explaining natural phenomena scientifically	3.03	75.80%
P2	Connecting science concepts with everyday life	3.07	76.70%
P3	Reading and understanding scientific graphs/tables	2.23	55.80%
P4	Drawing conclusions from experimental results	2.47	61.70%
P5	Designing simple experiments	3.10	77.50%
P6	Distinguishing scientific and non-scientific information	2.97	74.20%
P7	Using evidence to support opinions	2.30	57.50%
**Overall**	**Perceived scientific literacy**	**2.74**	**68.50%**

Students also reported relatively high perceived competence in distinguishing scientific from non-scientific information, with a mean of 2.97 (74.20%). In contrast, the lowest perceived competence was found in reading and understanding scientific graphs or tables, with a mean of 2.23 (55.80%). The ability to use evidence to support an opinion was similarly low, with a mean of 2.30 (57.50%). The ability to draw conclusions from experimental results obtained a mean of 2.47 (61.70%).

The overall mean was 2.74 out of 4.00, corresponding to 68.50%. The questionnaire document classified students’ perceived scientific literacy at the moderate level. Thus, at the level of self-reported competence, the highest percentages were associated with designing simple experiments (77.50%), connecting science concepts with everyday life (76.70%), and explaining natural phenomena scientifically (75.80%). The lowest percentages were found for reading and understanding graphs/tables (55.80%) and using evidence to support opinions (57.50%).

### Students’ demonstrated scientific literacy

The objective assessment consisted of 15 multiple-choice items and was administered to 30 junior high school students. The test assessed scientific literacy through contextual science problems covering environmental and ecological phenomena, including pollution, global warming, eutrophication, deforestation, water quality, plastic waste, biodiversity, pesticides, air quality, land use, biodegradable materials, ozone depletion, marine pollution, and mangrove ecosystems.

The test blueprint indicates that the items required cognitive processes ranging from C2 to C6. The assessed competencies included explaining scientific phenomena, designing scientific investigations, interpreting data, evaluating evidence, analyzing environmental effects, and designing experimental controls.

According to the official summary provided in the assessment document ( Table 4), the students obtained an average of 5.97 correct answers out of 15 items, corresponding to an average score of 39.80%. The median score was 40.00%, while the minimum and maximum scores were 20.00% and 73.30%, respectively.

**Table 4.** Descriptive statistics of the objective scientific literacy test.

Statistic	Result
Number of students	30
Number of items	15
Mean number of correct answers	5.97/15
Mean score	39.80%
Median	40.00%
Minimum	20.00%
Maximum	73.30%

Based on Table 5, the distribution of students across the four performance categories further shows that 12 students (40%) were classified as low, 12 students (40%) as moderate, and 6 students (20%) as high. No student reached the very-high category.

**Table 5.** Distribution of demonstrated scientific literacy.

Category	Score range	n	%
Low	<40%	12	40%
Moderate	40–59%	12	40%
High	60–79%	6	20%
Very high	80–100%	0	0%
**Total**		**30**	**100%**

The objective assessment therefore shows that 80% of the students were in the low or moderate categories, while the remaining 20% were in the high category. No student reached the very-high category.

### Comparison of perceived and demonstrated scientific literacy

To further examine the comparison between students’ perceptions and their demonstrated scientific literacy, the self-reported scores were compared with the average performance obtained from the corresponding objective-test items across four directly comparable domains. The comparison focused on scientific explanation, data/graph/table interpretation, conclusion drawing, and experimental/investigation design. For each domain, the demonstrated score was calculated from the percentage of correct responses to the items representing that domain, while the gap was expressed as the difference between the perceived and demonstrated scores. The results of this comparison are presented in Table 6.

**Table 6.** Descriptive comparison of perceived and demonstrated scientific literacy.

Domain	Perceived/Self-report (%)	Demonstrated Scientific Literacy (%)	Gap (Perceived − Demonstrated)
Scientific explanation	75.80	43.33	32.47
Data/graph/table interpretation	55.80	41.10	14.70
Conclusion drawing	61.70	21.65	40.05
Experimental/investigation design	77.50	42.48	35.03

The comparison presented in Table 6 shows a consistent discrepancy between students’ perceived and demonstrated scientific literacy across all four domains. In each domain, the perceived score was higher than the corresponding demonstrated score. The largest discrepancy was found in conclusion drawing, where students reported a perceived competence of 61.70%, whereas their demonstrated performance averaged only 21.65%, resulting in a 40.05-percentage-point gap. A similarly substantial discrepancy was observed for experimental/investigation design, with a perceived score of 77.50% compared with a demonstrated score of 42.48%, producing a 35.03-percentage-point gap.

For scientific explanation, students reported a perceived competence of 75.80%, while their average demonstrated performance was 43.33%, corresponding to a 32.47-percentage-point discrepancy. The smallest discrepancy occurred in data/graph/table interpretation, although a difference remained evident: the perceived score was 55.80%, compared with a demonstrated score of 41.10%, resulting in a 14.70-percentage-point gap. Notably, this domain also had the lowest perceived score among the four domains, yet its demonstrated performance was higher than that observed for conclusion drawing.

Overall, the findings indicate that students’ perceptions of their scientific literacy were consistently more favorable than their demonstrated performance on the objective assessment. The discrepancy was particularly pronounced for conclusion drawing and experimental/investigation design, suggesting that students’ confidence in these competencies was not fully reflected in their performance on corresponding assessment tasks. In contrast, the relatively smaller discrepancy in data/graph/table interpretation indicates a closer, although still incomplete, correspondence between perceived and demonstrated competence in this domain.

### Summary of the main findings

The main findings of the study are summarized in Table 7 to provide an integrated overview of students’ learning interest, project-based learning experience, perceived scientific literacy, demonstrated scientific literacy, and the discrepancy between perceived and demonstrated competence. The summary brings together the descriptive findings reported in the preceding sections and highlights the overall and domain-level patterns that are most relevant to the study’s research questions.

**Table 7.** Summary of the main findings.

Domain	Main finding
Science learning interest	Students reported generally positive interest in science learning, with enjoyment of science lessons receiving the highest score (95.00%).
Active participation	Asking questions during science lessons was the lowest interest-related indicator (65.00%).
Project-based learning experience	88.30% of students reported previous participation in project-based learning.
Project usefulness	88.30% perceived project-based learning as helpful for understanding science content.
Project-related difficulty	88.30% reported experiencing difficulties when completing project activities.
Project presentation	67.50% reported experience presenting project results.
Perceived scientific literacy	The overall perceived scientific literacy score was 68.50%, classified as moderate.
Strongest perceived domain	Experimental/investigation design received the highest perceived score among the four comparable domains (77.50%).
Lowest perceived domain	Data/graph/table interpretation received the lowest perceived score among the four comparable domains (55.80%).
Demonstrated scientific literacy	The overall demonstrated scientific literacy score was 39.80%.
Objective performance distribution	80% of students were classified in the low or moderate categories, while 20% were classified as high; no students reached the very-high category.
Overall perception–performance gap	The overall difference between perceived and demonstrated scientific literacy was 28.70 percentage points.
Largest domain-level gap	Conclusion drawing showed the largest discrepancy, with a 40.05-percentage-point gap between perceived (61.70%) and demonstrated (21.65%) competence.
Second-largest domain-level gap	Experimental/investigation design showed a 35.03-percentage-point gap between perceived (77.50%) and demonstrated (42.48%) competence.
Scientific explanation gap	Scientific explanation showed a 32.47-percentage-point gap between perceived (75.80%) and demonstrated (43.33%) competence.
Smallest domain-level gap	Data/graph/table interpretation showed the smallest discrepancy (14.70 percentage points), with perceived competence at 55.80% and demonstrated performance at 41.10%.

Taken together, the findings reveal a consistent pattern in which students’ perceived scientific literacy was higher than their demonstrated performance. The overall difference was 28.70 percentage points, while the domain-level analysis showed that the discrepancy was particularly pronounced in conclusion drawing and experimental/investigation design. In contrast, data/graph/table interpretation showed the closest correspondence between perceived and demonstrated competence. These findings provide an empirical basis for the subsequent discussion of why students’ perceptions may not fully correspond with their demonstrated scientific literacy and what this discrepancy implies for science instruction.

## Discussion

### The central finding: a consistent perception–performance discrepancy

The central finding of this study is the consistent difference between students’ perceived and demonstrated scientific literacy. Across the overall assessment and the directly comparable domains, students tended to perceive themselves as more capable than their objective-test performance indicated. This pattern is important because it shows that students’ perceptions and demonstrated abilities provide related but non-equivalent forms of evidence about scientific learning. The self-report questionnaire captures how students understand and judge their own capabilities, whereas the objective assessment provides evidence of what they can demonstrate when confronted with contextualized scientific problems. The two measures should therefore not be interpreted as interchangeable indicators of the same underlying performance.

This finding is consistent with the broader concern in self-assessment research that students’ judgments of their own competence do not always correspond closely with external measures of performance ( Carroll, 2020; González-Betancor et al., 2019). The discrepancy may be particularly relevant when the assessed competence involves complex reasoning rather than the recognition or recall of familiar knowledge. Scientific literacy requires students to interpret information, evaluate evidence, construct explanations, and make warranted decisions ( Valladares, 2021). These processes may be more difficult for students to judge accurately than more familiar forms of classroom learning.

The present findings should therefore be interpreted as evidence of a perception–performance discrepancy, rather than as proof that students systematically overestimated their abilities. Because the study used a descriptive comparison rather than an individual-level calibration analysis, the results do not establish the accuracy of each student’s self-judgment. Nevertheless, the consistent direction of the discrepancy across the comparable domains suggests that relying solely on students’ perceived competence may provide an incomplete picture of their scientific literacy. This supports the value of combining self-report evidence with direct performance assessment when diagnosing students’ learning needs.

### Why perceived competence may not correspond to demonstrated performance

One possible explanation for the observed discrepancy is the difference between familiarity with a learning activity and mastery of the reasoning processes required to perform it successfully. Students may have encountered scientific concepts, participated in classroom activities, or completed projects and consequently developed a sense that they are capable of performing related tasks. However, objective assessment requires them to transfer this knowledge to a particular problem, select relevant evidence, interpret representations, and determine an appropriate response. Perceived familiarity with a task may therefore be greater than the ability to execute the underlying reasoning process independently.

This distinction is especially relevant to scientific literacy because many of its components are procedural and epistemic rather than purely conceptual. Knowing what a scientific concept means does not necessarily imply being able to use that concept to explain an unfamiliar phenomenon. Similarly, having participated in an experiment does not necessarily mean that a student can identify an appropriate control condition or evaluate whether the evidence supports a causal claim. The objective assessment used in this study included contextualized problems requiring students to apply scientific knowledge and reasoning rather than simply recall information.

The findings can also be considered from a metacognitive perspective. Self-assessment requires students to judge the quality of their own knowledge and performance, and such judgments may be influenced by what students remember doing rather than by how successfully they can perform a comparable task under assessment conditions. This possibility is particularly relevant for junior high school students, who are still developing more sophisticated forms of metacognitive monitoring. The discrepancy observed here may therefore indicate that students need more opportunities to compare their expectations with evidence from their own performance.

Importantly, this does not imply that self-report data are uninformative. Students’ perceptions provide information that cannot be obtained from an objective test alone, particularly regarding their confidence, awareness of strengths and weaknesses, and understanding of their own learning. The value lies in examining the two sources together. A student who performs poorly but recognizes the difficulty may require a different instructional response from one who performs similarly but believes that the competency has already been mastered. Thus, the perception–performance comparison can contribute to a more differentiated diagnosis of learning needs.

### Domain-level discrepancies in scientific literacy

The domain-level analysis provides a more specific picture of the discrepancy than the overall comparison. Although students’ perceived competence exceeded demonstrated performance in all four comparable domains, the magnitude of the discrepancy varied considerably. This variation suggests that the perception–performance gap is not uniform across scientific literacy competencies. Instead, some forms of scientific reasoning appear to be more difficult for students to judge accurately than others.

The largest discrepancy occurred in conclusion drawing. This finding is particularly important because drawing a scientific conclusion requires more than identifying a plausible answer. Students need to determine whether a conclusion is warranted by the available evidence and distinguish between what the data directly support and what they may merely suggest. The relatively weak demonstrated performance in this domain may indicate difficulties in connecting evidence to a defensible conclusion. Students may recognize the general direction of a phenomenon while still struggling to determine which conclusion is sufficiently supported by the data. This distinction is central to scientific reasoning because scientific claims are expected to be justified by evidence rather than by plausibility alone.

The finding also helps explain why students’ self-perceptions may be more favorable than their demonstrated performance. In classroom contexts, “drawing a conclusion” may sometimes be experienced as reaching an answer at the end of an activity. In contrast, the objective assessment requires students to evaluate the relationship between information presented in the task and the conclusion that follows from it. The difference between these two interpretations of the task may contribute to the observed discrepancy.

From an instructional perspective, conclusion drawing should therefore be treated as an explicit reasoning process rather than merely the final step of an experiment. Students need opportunities to move systematically from evidence to interpretation and then to conclusion, while also being asked to justify why a particular conclusion is supported ( Mata-Pereira & da Ponte, 2017; Widjaja et al., 2021). Such practices can make the evidentiary basis of conclusions more visible and may help students develop a more accurate understanding of their own competence.

A similarly substantial discrepancy was observed in experimental/investigation design. Interestingly, this was the domain in which students expressed the strongest perceived competence among the four comparable domains, yet their demonstrated performance remained considerably lower. This contrast suggests that students may feel familiar with the general idea of conducting experiments without necessarily mastering the reasoning required to design or evaluate an investigation.

Scientific investigation design involves more than identifying an experimental activity. Students need to consider variables, comparison conditions, controls, procedures, and the type of evidence required to evaluate a claim ( Kuhn & Modrek, 2022; O’Keefe, 2023). These elements require students to reason about how an investigation can provide valid evidence rather than simply identify an activity that appears relevant to the problem.

The finding is particularly relevant in light of students’ reported experience with project-based learning. Most students indicated that they had previously participated in project activities and perceived such activities as useful for understanding science. However, the present study does not provide evidence that project-based learning caused either higher or lower scientific literacy performance. Rather, the findings suggest that participation in projects alone should not be assumed to guarantee mastery of scientific investigation reasoning.

Project-based learning can provide valuable opportunities for students to investigate authentic problems, collaborate, apply concepts, and produce meaningful outcomes ( Guo et al., 2020; Hamid et al., 2026; Swandi et al., 2023). However, its contribution to scientific literacy depends partly on the cognitive and epistemic processes embedded in the projects. A project may successfully produce a product or solve a practical problem while providing relatively limited opportunities for students to explicitly identify variables, evaluate evidence, justify causal claims, or assess the adequacy of an experimental design. This distinction has also been recognized in the literature discussed in the manuscript, which emphasizes that the educational value of project-based learning depends not simply on participation but on the quality of reasoning processes incorporated into project activities.

The implication is therefore not that project-based learning should be reduced, but that project activities should more deliberately incorporate scientific investigation reasoning. Students should be required to explain why a particular variable is controlled, why a comparison condition is necessary, what evidence would support a claim, and how an investigation could be improved. Such explicit reasoning could help narrow the distance between students’ familiarity with project activities and their demonstrated ability to design scientifically defensible investigations.

Scientific explanation also showed a substantial difference between perceived and demonstrated competence. Students appeared relatively confident in their ability to explain scientific phenomena, but this confidence was not fully reflected in their performance on the corresponding objective tasks.

One possible interpretation is that students may associate scientific explanation with describing a phenomenon or recalling a relevant concept. However, an adequate scientific explanation requires a meaningful connection between the phenomenon and the scientific mechanism that accounts for it. In other words, students need to move beyond stating what happens toward explaining why or how it happens using scientifically appropriate concepts.

The contextual nature of the objective assessment is important here. The test presented students with environmental and ecological situations involving pollution, land use, plastic waste, pesticides, water systems, and other phenomena. Such contexts require students to transfer scientific knowledge to situations that may not be identical to those encountered during classroom instruction ( Khishfe, 2019; Kulasegaram et al., 2017).

The discrepancy therefore suggests that instruction may need to provide more opportunities for students to construct explanations in unfamiliar contexts. Rather than asking students only to identify concepts or describe familiar phenomena, teachers can ask them to articulate causal relationships, connect observations to scientific principles, and defend their explanations using available evidence. This would make the distinction between knowing a concept and using the concept to explain a phenomenon more explicit.

Data/graph/table interpretation presented a different pattern. Among the four domains, it showed the closest correspondence between students’ perceptions and their demonstrated performance. Although the correspondence was not complete, the relatively smaller discrepancy suggests that students’ perceptions of their ability in this area may have been somewhat more closely aligned with what they could demonstrate.

This finding is noteworthy because students also reported comparatively lower confidence in this competency in the self-report questionnaire. In contrast to the other domains, students did not appear to perceive themselves as highly capable interpreters of scientific representations. Their objective performance was also limited, producing a narrower distance between perception and performance.

One possible explanation is that students may have more direct awareness of the difficulty of reading graphs and tables. Unlike broader competencies such as “explaining science” or “designing an experiment,” interpreting a graph or table presents a relatively visible task demand. Students may recognize when they have difficulty extracting information from a representation, which could contribute to a closer correspondence between perceived and demonstrated competence.

However, the relatively smaller discrepancy should not be interpreted as evidence of strong mastery. The objective performance indicates that interpreting scientific representations remains an area that warrants instructional attention. The important distinction is that the issue appears to involve both performance difficulty and greater awareness of that difficulty, rather than the larger perception–performance discrepancy observed in the other domains.

### Interest and project-based learning experience: engagement is not sufficient

The findings concerning students’ interest in science and their experience with project-based learning provide useful context for interpreting the scientific literacy results. Students generally expressed positive attitudes toward science and reported substantial exposure to project-based learning. They also viewed project activities as useful for understanding science. At the same time, the objective assessment revealed limitations in the scientific reasoning competencies examined in this study.

This combination suggests that affective engagement and learning exposure should not be equated with demonstrated scientific literacy ( Fortus et al., 2022; Lin et al., 2012). Students can enjoy science, recognize its relevance, collaborate with peers, and participate actively in projects while still experiencing difficulty with higher-order reasoning tasks. Interest is therefore an important foundation for engagement, but it does not automatically translate into the ability to interpret evidence, construct conclusions, or design scientifically appropriate investigations ( Wu et al., 2018; Wu & Wu, 2020).

The same principle applies to project-based learning. The students’ reported experience indicates that projects are already part of their learning environment, but the demonstrated performance suggests that participation may need to be accompanied by more explicit attention to scientific reasoning. This is consistent with the argument that project-based learning can support scientific literacy when students are required to engage meaningfully with evidence and reasoning throughout the project process ( Jayati & Fitriani, 2025; Markula & Aksela, 2022; Sari et al., 2025).

An important implication is that science instruction should avoid treating engagement and performance as interchangeable outcomes. Enjoyment of science and participation in projects are valuable because they create opportunities for learning, but those opportunities need to be structured so that students repeatedly practice the specific reasoning processes that scientific literacy requires. This distinction may be particularly important in junior high school, where students are increasingly expected to move from recalling scientific information toward interpreting evidence, evaluating explanations, and reasoning about investigations.

### Implications for science instruction

The findings have several implications for science instruction. First, self-reported competence should not be used as the sole basis for diagnosing scientific literacy. Students’ perceptions provide useful information about how they understand their own abilities, but these perceptions should be complemented by tasks that require students to demonstrate scientific reasoning. Combining the two forms of evidence can help teachers identify situations in which students are confident but still require substantial support, as well as situations in which students may underestimate abilities they can actually demonstrate.

Second, instruction should make evidence-based conclusion drawing more explicit. Given the pronounced discrepancy in this domain, students should be given repeated opportunities to distinguish observations from interpretations, identify which evidence supports a claim, and evaluate whether a conclusion is warranted. Rather than treating conclusion drawing as the final sentence in a laboratory report, teachers can make the reasoning chain visible by asking students to explain how the evidence leads to the conclusion.

Third, experimental and investigation reasoning should receive more explicit attention within practical and project-based activities. Students should not only perform procedures but also explain why an investigation is designed in a particular way. Questions concerning variables, controls, comparison groups, evidence, and alternative explanations can be integrated into project work without reducing the authenticity of the activity.

Fourth, scientific explanation should be developed through tasks that require students to apply concepts to unfamiliar phenomena. This can help students move from descriptive responses toward causal explanations and strengthen the transfer of scientific knowledge across contexts. Finally, the relatively closer correspondence in data/graph/table interpretation suggests the potential value of helping students develop greater awareness of their own performance through formative assessment. When students receive opportunities to compare their predictions of performance with actual results and then reflect on the reasons for discrepancies, assessment itself can become a tool for developing metacognitive awareness. Such practices may help students gradually develop more realistic judgments of their scientific capabilities.

### Limitations and directions for future research

Several limitations should be considered when interpreting these findings. First, the study involved a relatively small group of junior high school students from two schools in Luwu Regency, South Sulawesi, selected through purposive sampling. The findings therefore provide a contextual description rather than a basis for broad statistical generalization to junior high school students in other regions.

Second, the study used a descriptive comparative design. The observed discrepancy between perceived and demonstrated scientific literacy should not be interpreted as evidence of a causal relationship between students’ perceptions and their performance, nor should it be used to evaluate the effectiveness of project-based learning. The findings describe patterns within the participating students and their learning context.

Third, the self-report questionnaire and objective assessment were complementary rather than parallel instruments. They differed in response format, scoring procedure, and measurement approach. Consequently, the comparison was intentionally descriptive rather than a psychometric test of equivalence between two forms of the same instrument.

Fourth, although the questionnaire included seven dimensions of perceived scientific literacy, only four domains had sufficiently direct representation in the objective assessment to support domain-level comparison. Other dimensions, such as connecting science concepts with everyday life, distinguishing scientific from non-scientific information, and using evidence to support opinions, were retained as part of the self-report profile but were not treated as directly equivalent objective-test domains. This approach reduces the risk of overstating correspondence between constructs that were not explicitly measured in parallel.

Future studies could address these limitations by involving larger and more diverse samples and by developing parallel self-report and performance measures for the same scientific literacy competencies. Individual-level paired data could also allow researchers to examine calibration more directly by determining whether students who perceive themselves as highly competent actually perform consistently at that level. Longitudinal or intervention studies could further investigate whether explicit instruction in evidence-based reasoning, experimental design, and reflective self-assessment can reduce the perception–performance discrepancy over time.

## Conclusion

This study examined junior high school students’ scientific literacy from two complementary perspectives: perceived competence reported through a self-report questionnaire and demonstrated competence assessed through an objective scientific literacy test. The findings indicate that students generally perceived themselves as moderately capable in scientific literacy, while their performance on the objective assessment revealed more substantial difficulties in demonstrating scientific reasoning. More importantly, the comparison across four directly comparable domains showed that perceived competence was consistently higher than demonstrated performance.

The domain-level findings further indicate that the discrepancy was not uniform across scientific literacy competencies. The largest divergence was observed in conclusion drawing, followed by experimental/investigation design and scientific explanation, whereas data/graph/table interpretation showed the closest correspondence between students’ perceptions and demonstrated performance. These patterns suggest that students may not always have an accurate understanding of the extent to which they can apply scientific reasoning, particularly when tasks require them to use evidence, justify conclusions, or reason about the design of an investigation.

The study therefore highlights the value of treating self-reported perceptions and objective performance as complementary rather than interchangeable sources of evidence. Students’ confidence and perceptions can provide useful information about their learning, but these perceptions need to be considered alongside direct evidence of what students can demonstrate. For science education, the findings point to the need for learning experiences that explicitly strengthen evidence-based reasoning, scientific explanation, investigation design, and reflective assessment of students’ own performance. Project-based and active learning activities may provide meaningful contexts for this work when they deliberately incorporate these scientific reasoning processes.

Overall, examining the perception–performance gap provides a more nuanced diagnosis of students’ scientific literacy than relying on either self-report or objective assessment alone. Future research involving larger samples, parallel measures of perceived and demonstrated competence, and individual-level calibration analysis could further clarify how students develop accurate judgments of their scientific abilities and how instruction can support closer alignment between what students believe they can do and what they are able to demonstrate.

## Ethical consideration and informed consent

The study was approved by the Institutional Review Board at Universitas Bosowa on 4 July 2025 (Reference No. 174b/1.02/DRIPM-UNIBOS/VII/2025). The student participants were minors aged approximately 13–14 years. In accordance with the ethical procedure reviewed and approved by the Institutional Review Board, verbal informed consent was obtained from the parents or legal guardians of the participating students. Parents or legal guardians were informed verbally about the study, including its purpose, procedures, and their children’s participation. Verbal consent was used because many parents or legal guardians lived at considerable distances from the participating schools and had limited access to written or electronic communication, making the collection of physical or electronic consent forms impractical. The Institutional Review Board reviewed the study protocol and considered the consent procedure appropriate for the study context. Permission to conduct the study was obtained from the participating schools and school principals, and the students voluntarily agreed to participate. Participation was voluntary, and students were informed that their responses would be treated confidentially and used solely for research purposes.

## AI statement

The authors stated that generative artificial intelligence tools were used in a limited manner to assist with language editing, organization of academic writing, clarity improvement, and image-quality enhancementimprovement. The conceptual framework, research design, data analysis, interpretation, and final conclusions were developed entirely by the authors.

## Data Availability

The data supporting the findings of this study are available in figshare:
• Scientific Literacy Test Instruments and Results. DOI: https://doi.org/10.6084/m9.figshare.33296193 ( Megawati, 2026b)
• Questionnaire Profile and Science Learning Needs. DOI: https://doi.org/10.6084/m9.figshare.33296190 ( Megawati, 2026a). Data are available under the terms of the Creative Commons Attribution 4.0 International (CC-BY 4.0) license.
