# Supplementary material for: Bridging the Perception–Performance Gap in Scientific Literacy: Evidence from Junior High School Students’ Self-Reports and Objective Assessment
Source: F1000Res. 2026 Sep 11;15:1528. [Version 1] doi: 10.12688/f1000research.189469.1 (PMC13736063; doi:10.12688/f1000research.189469.1)
Supplement: Peer review history

# Reviewer report: Reviewer response for version 1

**DOI:** 10.5256/f1000research.209113.r522234

**Published:** 2026-11-04

**Authors:** Citra Ayu Dewi

**Affiliations:** 1 Chemistry Education, Universitas Pendidikan Mandalika, Mataram, West Nusa Tenggara, Indonesia

**Introduction:**

 This introduction does not explain the research problem, the importance of the research conducted, the contribution of the research, the novelty of this research is explained, it is necessary to mention the problem formulation, and the research objectives are not explained in this article.

**Methods:**

 The following should be included/amended:

 1.    The article needs to explain more clearly and in enough depth the research approach of the study.

 2.    The methods should be adequately described to show how the research was conducted to improve its transparency and trustworthiness. For example, the use of the data-gathering methods needs to be justified, and their appropriateness needs to be substantiated in the literature.

 3.    How was the sample size determined? Was it considering the population? If yes, what is the population size?

 4.    This section could be improved by excluding results, such as the results of the collected sample, like in Table 1, etc.

 5.    Similarly, it needs to be clear how the data analysis methods used in the paper are appropriate for analysing the data obtained. It can be extended to explain the stages of data analysis, including the measurement and structural models.

 6.    It is also suggested to discuss the reliability and validity of the study outcomes. It is also essential to address the study’s limitations and provide a comprehensive discussion of the expected benefits of the proposed new teaching practice.

**Result and Discussion:**

 The comparisons of the current findings with the findings of previous research are not discussed. Furthermore, elaborations regarding the rejected hypotheses are also not included. Thus, the discussions could be summarized by stating clear answers to the research questions.

**Conclusion:**

 1.    The aims and objectives of the study are not summarized in the conclusion. Furthermore, the main findings are not explicitly stated.

 2.    The study’s contribution must be clearly articulated in this section.

 3.    The section needs to include recommendations for practitioners based on the findings, if appropriate.

Is the work clearly and accurately presented and does it cite the current literature?

Partly

If applicable, is the statistical analysis and its interpretation appropriate?

Partly

Are all the source data underlying the results available to ensure full reproducibility?

Partly

Is the study design appropriate and is the work technically sound?

Partly

Are the conclusions drawn adequately supported by the results?

Partly

Are sufficient details of methods and analysis provided to allow replication by others?

Partly

Reviewer Expertise:

Recommendation: Major Revision

I confirm that I have read this submission and believe that I have an appropriate level of expertise to confirm that it is of an acceptable scientific standard, however I have significant reservations, as outlined above.

# Reviewer report: Reviewer response for version 1

**DOI:** 10.5256/f1000research.209113.r531525

**Published:** 2026-11-02

**Authors:** Ruth A. Ortega-Dela Cruz

**Affiliations:** 1 University of the Philippines Los Banos, Laguna, Calabarzon, Philippines

Dear Editor,

 Thank you for the opportunity to review this manuscript.

 The article addresses a relevant and worthwhile issue in science education, particularly the relationship between students perceived and demonstrated scientific literacy. The introduction provides a reasonable theoretical foundation, and the research design is generally aligned with the study's descriptive purpose. However, several aspects require improvement to strengthen the article's conceptual clarity, methodological rigor, and contribution to the field.

 My comments and recommendations are as follows:

1. Introduction. The research gap is stated, but the novelty and specific contribution of the study remain insufficiently clear. Explicitly identify the novelty of investigating these dimensions among junior high school students in the Indonesian context. Some claims about the relationship between project-based learning and scientific literacy need stronger supporting evidence.
2. Research design. The descriptive comparative quantitative design is appropriate for the stated objectives. However, the term comparative may imply statistical comparisons between groups or variables, whereas the study primarily describes and interprets two sources of evidence. The relationship between the research questions and the analytical procedures needs to be more explicit. Consider using descriptive quantitative design with a comparative analysis of perceived and demonstrated competence. Explain how each research question is addressed by the corresponding analytical procedure. Clarify whether the study examines individual-level discrepancies or only compares aggregate scores. This distinction is important.
3. Participants and sampling. The sample consists of only 30 students from two schools, which limits the generalizability of the findings. The rationale for selecting the two schools and the participants through purposive sampling is insufficiently explained. The study does not clearly indicate how many students came from each school or their respective grade levels. Provide the number of participants from each school and their grade distribution. Explain the inclusion criteria and reasons for selecting the participating schools. Acknowledge the small sample size and sampling limitations when interpreting the findings. Avoid making broad claims about junior high school students based on this relatively small sample.
4. Ethical considerations. The ethical approval information is clearly presented. However, obtaining verbal parental consent for research involving minors requires particularly careful documentation. Clarify whether student assent was obtained separately from parental consent.
5. Research instruments and validity. Provide sample items from both instruments to illustrate how scientific literacy was assessed. Explain the validation procedure, scoring formula, and basis for the 75% validity threshold. Report reliability evidence, such as Cronbach's alpha or another appropriate reliability estimate, where applicable.
6. Please conduct thorough language editing to improve conciseness and academic flow. Remove unnecessary repetition, especially in the Introduction.

These revisions would greatly enhance the manuscript’s clarity, depth, and overall scholarly contribution.

 Thank you for considering these suggestions.

 Very truly yours,

 Ruth Ortega-Dela Cruz, PhD

 Professor

 University of the Philippines Los Banos

Is the work clearly and accurately presented and does it cite the current literature?

Yes

If applicable, is the statistical analysis and its interpretation appropriate?

Yes

Are all the source data underlying the results available to ensure full reproducibility?

Yes

Is the study design appropriate and is the work technically sound?

Partly

Are the conclusions drawn adequately supported by the results?

Yes

Are sufficient details of methods and analysis provided to allow replication by others?

Partly

Reviewer Expertise:

Education

I confirm that I have read this submission and believe that I have an appropriate level of expertise to confirm that it is of an acceptable scientific standard, however I have significant reservations, as outlined above.

# Reviewer report: Reviewer response for version 1

**DOI:** 10.5256/f1000research.209113.r522235

**Published:** 2026-09-18

**Authors:** Bayu Fitra Prisuna

**Affiliations:** 1 Pontianak State Islamic Institute, Pontianak, West Kalimantan, Indonesia

The authors have tackled a very important and timely topic with great clarity. I thoroughly enjoyed reading this article and appreciate the novel insights presented in the discussion section.

 Several references are outdated and should be replaced with more recent literature to reflect the current state of the art.

 Thanks

Is the work clearly and accurately presented and does it cite the current literature?

Yes

If applicable, is the statistical analysis and its interpretation appropriate?

Yes

Are all the source data underlying the results available to ensure full reproducibility?

Yes

Is the study design appropriate and is the work technically sound?

Yes

Are the conclusions drawn adequately supported by the results?

Yes

Are sufficient details of methods and analysis provided to allow replication by others?

Yes

Reviewer Expertise:

measurement and evaluation

I confirm that I have read this submission and believe that I have an appropriate level of expertise to confirm that it is of an acceptable scientific standard.
